# A Pilot Randomized Controlled Trial to Examine the Impact of a Therapy Dog Intervention on Depression, Mood, and Anxiety in Hospitalized Older Adults

**DOI:** 10.3390/healthcare13151819

**Published:** 2025-07-25

**Authors:** Nancy R. Gee, Lisa Townsend, Erika Friedmann, Sandra Barker, Megan Mueller

**Affiliations:** 1 Center for Human-Animal Interaction, Department of Psychiatry, Virginia Commonwealth University School of Medicine, Richmond, VA 23298, USA; lisa.townsend@vcuhealth.org (L.T.); sbbarker@vcu.edu (S.B.); 2 Department of Organizational Systems and Adult Health, University of Maryland Baltimore School of Nursing, Baltimore, MD 21201, USA; friedmann@umaryland.edu; 3 Cummings School of Veterinary Medicine, Tufts University, North Grafton, MA 01536, USA; megan.mueller@tufts.edu

**Keywords:** animal-assisted intervention, mental health, well-being, anxiety, depression, mood

## Abstract

**Background/Objectives**: Aging adults are at an increased risk of depression, anxiety, and poor mood. Research indicates that interacting with companion animals may benefit vulnerable older adults’ mental health. **Methods**: This study randomly assigned 44 medical inpatients (59+ years) to one of three 20 min daily conditions over 3 days: interactions with a dog and handler (AAI: animal-assisted intervention), handler-only control (CC: conversational control), or usual care (UC). Participants were measured at baseline, daily pre/post-intervention, on day 5 post-intervention, and at 1- and 6-month follow-ups. **Results**: The results show a significant change in trajectory for the AAI group from pre- to post-intervention for anxiety and mood. Study satisfaction was significantly better in the AAI group than in the UC condition. Depression scores did not change significantly. The effects were not sustained from day 1 to day 5 or to either follow-up. **Conclusions**: The results suggest that short-term interactions with dogs may provide immediate improvements in anxiety and mood, and dog presence adds value above that of the handler alone.

## 1. Introduction

There is no question that the number of older adults is increasing. Globally, their numbers are expected to grow twofold by 2050 [1]; in some countries, the expectation is that they will quadruple, reaching 2.2 billion by the late 2070s [2]. Despite losses in the over-65 cohort as a result of the COVID-19 pandemic, their population percentage in some regions is also expected to double by 2050 [3]. It is well established that aging brings with it diminished physical and cognitive functioning and a reduced sense of overall well-being [4]. Health and satisfaction with quality of life are both negatively impacted, either directly or indirectly, by the growth of social isolation and accompanying feelings of loneliness and depression. The U.S. Surgeon General [5] has pronounced these conditions as reaching epidemic proportions. In a recent longitudinal study, a reduction in well-being alongside an increase in anxiety was found to be associated with the occurrence of the COVID-19 pandemic [6]. It is also well known that depression is highly prevalent among older adults, with a prevalence rate of about 35% [7], and is commonly associated with lower quality of life, increased comorbidities, and worsening of chronic illness [8].

There is now an accumulation of evidence indicating that interacting with a companion animal has beneficial effects on human mental health. The quality of the science and the strength of that evidence tends to be superior for studies involving humans interacting with animals in an intervention setting than for studies focusing on pet ownership, because pet ownership studies usually suffer from a selection bias in that people come to the studies having preselected their own pet (or choosing not to have a pet) rather than allowing a researcher to randomly assign them to a pet ownership category [9]. For example, a recent systematic review of 37 studies involving canine-assisted interventions revealed that interacting with a dog in an intervention setting improves mental health outcomes in higher education students, particularly anxiety and stress, and it also has the social benefit of encouraging communication and shared experience [10]. Much work in human–animal interaction research has focused on the role of animals in mitigating mental illness, such as depression and anxiety [11]. In fact, the involvement of animals in the treatment of mental disorders is becoming more common and has been detailed in a recent book on the subject [12].

In a systematic review of the evidence, Gee & Mueller [9] examined 145 studies that focused on the impact or association of companion animals on a number of outcome variables for older adults. They report that the evidence is clearer, of higher quality, and more consistent for studies involving animal interaction within intervention and/or therapeutic settings than for those addressing the relationship of pet ownership to the various outcome variables. Specifically, they identified 17 studies on the effect of animal-assisted interactions or interventions (AAIs) on depression in older adults. The majority of the results from those studies indicated that when older adults interact with companion animals in an AAI, depression scores are reduced, but they also warn that these results must be interpreted with caution as they are from a heterogenous array of treatments, methodologies, and measures. For example, in one study, participants were assigned to care for crickets over the course of an 8-week intervention [13]; in another study, participants were assigned a three-month exposure to a canary, a plant, or nothing [14], while another study involved an 11-week intervention in which participants were assigned to dog-assisted therapy or a human therapist only [15]. It is worth noting that these studies tend to involve a recurring interaction over a period of time (e.g., 8 weeks, 3 months, or 11 weeks) rather than a one-time or a very short-term interaction over a few days. This raises the question as to whether a short-term intervention or a longer intervention period is required to see movement in depression scores. Additionally, these studies included a variety of animals (e.g., crickets, canaries, and dogs), raising the possibility that a variety of animal species may support improvements in depression when included in these types of interventions. However, at this point, the literature contains more information on the impact of dog-based programs than other species for older adults.

A recent review and meta-analysis of dog visitation programs for older adults likewise points to heterogeneity of methodology and sample size in the literature but nevertheless reports large beneficial effects of interactions with dogs for depressive symptoms in older adults [16]. In a systematic review of the literature, eight studies were identified as involving interventions with dogs for residents with dementia in nursing homes, which had “outcomes related to depression and mood.” Here again we see a variety of methodologies used, and six of the eight studies used different assessment instruments. Even so, six of the eight studies reported significant positive effects of dog interaction, while the remaining two studies reported positive tendencies [17]. Yet another systematic review and meta-analysis examined 47 studies (57.4% of which involved dogs, 10.6% horses, and the remaining involved various other species, including robotic animals) with the goal of providing a guide to evidence-based practice [18]. They calculated effects for depression from 14 studies and reported a significant decrease in depression among older adults. Among the 26 quasi-experimental studies examined, they also reported a number of positive mood, or mood-related, outcomes. For example, one study showed significant increases in smile episodes when visiting with a dog [19], while another study reported improvements in positive and negative mood following visits from a dog [20]. It is possible that mood as a measure is more immediately reactive than depression to any potential beneficial effects of interacting with a dog because the Phelps [20] study involved only six brief 5–10 min interactions with a dog.

Although anxiety is frequently comorbid with depression [21,22], it is important to examine it separately because it also represents a distinct construct and has different diagnostic criteria [23]. In their systematic review of the evidence, Gee and Mueller [9] identified eight studies that specifically assessed anxiety and anxiety-related constructs or behaviors associated with anxiety. The results reported have mixed outcomes, with some studies showing, for example, that AAI sessions reduced anxiety in advanced heart failure patients [24] and Alzheimer’s patients [25], whereas other studies showed that AAI did not reduce anxiety in residents of long-term care facilities [26]. Several studies did show that AAI reduced behavior problems that may be related to anxiety [9]. For example, Barker and colleagues [27] showed that AAI involving a dog reduced fear in patients waiting to undergo electroconvulsive shock therapy. As was the case with the depression results presented above, it is important to keep in mind that the studies include a wide variety of methodologies, intervention types, and durations, as well as different measures, and primarily involve dogs, so we need to be cautious when making interpretations of the overall pattern of results.

Potentially, the most vulnerable among the older adult population, those who are hospitalized due to injury or exacerbation of illness, are at a substantially elevated risk of depression and anxiety [28]. The current study was designed to examine the impact of a dog visitation intervention on depression, mood, and anxiety in a group of older adults hospitalized for a minimum of five days. Participants were randomly assigned to receive visits from a dog and their human handler (AAI) or from the human handler without the dog (CC, a conversational control condition), or usual care (UC), for 20 min a day over a three-day intervention, with a 1- and 6-month follow-up. Based on previous research, we predicted that anxiety and depression would be reduced and mood would be elevated in the AAI condition compared to the UC condition from pre- to post-intervention. We predicted that scores in the CC condition would improve on all three measures, but not with the same magnitude as for the AAI condition. The inclusion of longitudinal follow-up measures, used infrequently but recommended for this type of research [29], will allow us to assess whether any effects of the dog intervention remain over time. Because our inclusion of longitudinal follow-up measures is exploratory, we are not able to make any specific predictions regarding the durability of the effects.

## 2. Materials and Methods

The methods employed in this study were developed specifically for use in this and similar studies conducted in the same location. Other published papers have resulted from related research (e.g., [29,30]).

### 2.1. Design

In this randomized controlled trial, the participants were randomly assigned to one of three treatment conditions: (1) animal-assisted intervention—visits from a therapy dog and their handler (AAI); (2) conversational control—visits from the handler without their dog (CC); or (3) usual care—usual care in a hospital setting (UC). This independent variable was delivered between participants (each participant only received one condition) and within participants (repeated assessments over the intervention and at 1- and 6-month follow-up). The primary outcome variables were anxiety, depression, and mood. The random assignment was generated using randomizer.org (accessed 19 September 2021). Participants were stratified by dog ownership (owner vs. non-owners) and were randomly assigned into one of the three conditions in blocks of six. EF, who had no contact with participants, generated all random assignments and placed them into individual envelopes. NG opened each envelope once a participant was successfully enrolled in the study. This process prevented experimenters from being aware, in advance, of individual condition assignments.

**Figure 1:**
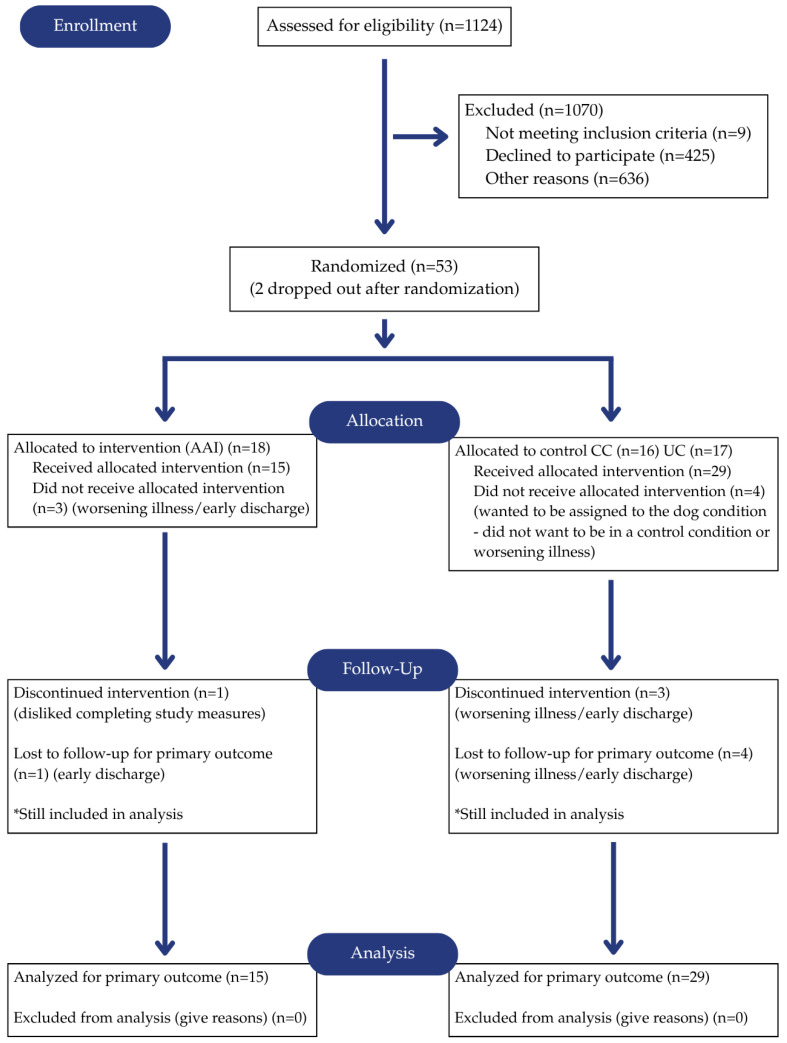
CONSORT 2025 [31] Flow Diagram showing the flow of participants through initial identification to enrollment, allocation, follow-up, and analysis in the study. * Indicates that participants were included in analyses.

The Consort Flow Diagram is presented in Figure 1. For brevity of presentation, we combined our two control conditions (CC and UC) on the right side of the diagram. All appropriate numbers are included in the diagram.

### 2.2. Participants

The sample comprised 44 participants, of which 50% were females (n = 22). Half (n = 22) were married, while 22.7% (n = 10) were widowed, 13.6% (n = 6) were divorced, and 13.6% (n = 6) were single/never married. Eighty percent (n = 35) of the sample identified as White, with twenty percent (n = 9) being Black/African American. Eighty-three percent (n = 35) had attended at least some college classes and most (74.4%, n = 32) were retired. The average age of the sample was 70.82 years (SD = 8.41); in the AAI condition, it was 71.80 (SD = 9.25), in the CC condition, it was 66.47 (SD = 6.20), and in the UC condition, it was 74.43 (SD = 7.98). Nineteen participants (43.2%) currently lived with a dog, and all participants were currently hospital in-patients.

### 2.3. Therapy Dogs and Handlers

All dogs who were part of this study were privately owned and were registered therapy dogs meeting the requirements of the Center for Human-Animal Interaction Dogs on Call (DoC) program at Virginia Commonwealth University [32]. The DoC program, fully detailed in Barker et al., 2019 [33], is evidence-based and provides complementary therapy to patients, staff, visitors, and students within VCU and the VCU Health System. Fourteen DoC dogs and fifteen human handlers (one dog had two handlers) were involved in this study. The average age (of those handlers sharing their age (n = 9)) was 60.55 years. Two of the fifteen handlers were male, fourteen were white, and one was white/Hispanic. There were 14 dogs involved in the study, including 2 Golden Retrievers, 2 Labrador Retrievers, 6 mixed breeds (Golden/Poodle, Maltese/Poodle, Flat Coated Retriever/Border Collie, German Shepherd/Australian Shepherd, Terrier and unknown) and 1 each of Miniature Poodle, Shih Tzu, Wire Hair Dachshund, and Miniature Schnauzer. They ranged from 3 to 11 years of age, 5 to 100 pounds in weight, and 11 to 29 inches in height.

Dog Requirements: All DoC dog–handler teams are required to hold a current registration with either Pet Partners or Alliance of Therapy Dogs (the latter group must also hold an American Kennel Club Canine Good Citizen title). Before being admitted to the program, they are evaluated in the VCU Health (VCUH) System by personnel of the Dogs on Call program. On an annual basis, handlers must provide for their dogs updated vaccination records, a negative fecal test, and evidence of a veterinary wellness exam. The dogs must be clean and odor-free, their nails must be trimmed and smooth, and they must wear an appropriate collar or harness in addition to their DoC vest. They must be kept on a 4-foot (or shorter) leash at all times.

Handler Requirements: All DoC handlers must first become VCUH volunteers, in which capacity they are subject to a complete background check and are tested for evidence of immunity to a variety of human diseases. Additionally, they must provide proof of annual influenza and COVID-19 vaccinations. As both VCUH volunteers and DoC team members, handlers participate in periodic online and in-person trainings, orientations, and observations, including Health Insurance Portability and Accountability Act (HIPAA) training, which focuses on protecting sensitive patient health information. Handlers participating in this study must also receive Community Engaged Research training described elsewhere [34]. Three methods of identification are required at all times for DoC handlers on every visit: their VCU Health photo identification cards, their Pet Partners or Alliance of Therapy Dogs identification cards, and their DoC polo shirt. Their dog’s leash must always be in their hand, and they must carry and use hand sanitizer approved by VCUH. During this study, handlers dispensed hand sanitizer at the beginning and conclusion of each interaction.

### 2.4. Measures

**Figure 2:**
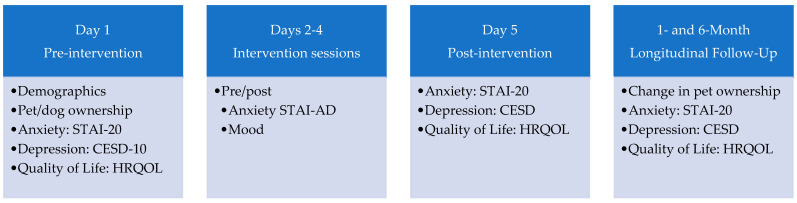
Delivery of Outcome Measures. STAI = State Trait Anxiety Inventory; STAI-20 is the 20-item version of the STAI, and STAI-AD is the five-item version of the STAI. CESD = Center for Epidemiological Studies Depression. HRQOL = Health-Related Quality of Life.

The Brief Interview for Mental Status (BIMS) [35] was used to screen for cognitive impairment. The BIMS has acceptable reliability (Cronbach’s alpha = 0.77) in evaluating attention, orientation, and recall in clinical settings, as well as good convergent and divergent validity. Intact cognitive functioning is indicated by scores of 13 or higher. Figure 2 displays the study outcome measures and the timing of the delivery of those measures, with descriptions of each below.

Demographics: Basic demographic information, including gender, age, race/ethnicity, education level, and marital and employment status, was collected from those participants who agreed to share this information.

Pet Ownership(s): A record was made of participant responses regarding previous and current pet ownership (including type and number of companion animal(s)).

Change in Pet Ownership: A 1-item question in the 1- and 6-month longitudinal follow-up inquired about any changes in the number of pets owned and an optional comment on the change.

Health-Related Quality of Life (HRQOL): The Centers for Disease Control and Prevention Core HQRL module, which consists of four items rating the impact of general physical and mental health on well-being, was measured on days 1 and 5. It has been extensively utilized with older adult populations [36,37].

Anxiety: Anxiety is “an emotion characterized by apprehension and somatic symptoms of tension in which an individual anticipates impending danger” [38]. This construct was measured using the 20 item State-Trait Anxiety Inventory (STAI-20) [39] and 5-item shortened scale of State-Trait Anxiety Inventory for Adults (STAI-AD) [40]. The STAI-AD has sound psychometric properties comparable to the long form, with Cronbach’s alphas ranging from 0.82 to 0.94.

Depression: Depression is a negative affective state that includes persistent feelings of sadness, loss of interest in activities, and a range of other symptoms that may interfere with daily life [41]. This construct was measured using the 10-item Center for Epidemiological Studies Depression short form (CESD-10) scale, which has been validated for use in older adults [42,43].

Mood: Mood is simply a person’s current emotional state of mind or feeling, and it was measured using the Smiley Face Assessment Scale, which depicts five emoticons from very sad to very happy [44]. This measure was used to provide a quick assessment of current mood pre/post-intervention.

Study Satisfaction Survey: On the 1-month longitudinal follow-up, participants were asked to report their satisfaction with the intervention they received on a scale from 1 (not at all) to 5 (a great deal). Those in the AAI and CC sessions were also asked an open-ended question that measured which aspects of the interaction they liked or did not like.

### 2.5. Procedure

Four locations within VCUH where older adults were most likely to be inpatients were identified: (1) Critical Care Hospital, (2) North Hospital, (3) Main Hospital (all acute care units), and (4) the VCUH Affiliate physical rehabilitation facility, Sheltering Arms Institute. VCUH units such as Cardiology, Acute Care Oncology, Acute Care Surgery, Intensive Care Units (Surgical, Cardiac, Neuroscience, Orthopedics), and rehabilitation units (Neurology, Multi-Specialty and Spinal Injury units) at Sheltering Arms Institute were targeted for participant recruitment.

Power: Power was calculated for the longitudinal outcome of loneliness. Similar power was required for depression, social support/isolation, HRQOL, and mood. Linear mixed models use all data and do not discard cases with missing data; this technique also has the advantage of modeling for dependence among multiple measures on the same cases. Typically, we see very high intercorrelations among multiple measures of mood over several weeks within individual; over the short term, pre- and post-intervention, and at 4 days, we expect high correlations. Since this is a pilot study, the ranges of values for the outcomes in this population are among the aims of the study. The rule of thumb for pilot studies is 12 cases per group [45]; we aimed for 20 per group in an effort to provide information that would allow for a better refinement of sample size calculations for a larger, outcome-focused randomized clinical trial. Our recruitment rate for this study was very low [45], so we ended up with 14 participants per group, exceeding the rule of thumb but falling short of our target.

Recruitment: In advance of study team visits, healthcare staff identified patients as potential participants (based on age and precautions which would prevent them from interacting with others, such as being COVID-19-positive or isolation status); upon arrival, recruiters approached these individuals. Using allocation concealment methods (described in the design section) ensured that recruiters were blind to group assignment during the enrollment process. The participant enrollment rate was under 5%; more than 1100 patients were assessed for eligibility to produce the 44 participants. Townsend et al. (2024) [29] report the details of recruitment feasibility for this study. Recruitment began in November of 2021 and ended May 2023, a period of 18 months.

Inclusion/Exclusion Criteria: There were five inclusion criteria in this study: participants had to speak English; be aged 59 or older; be projected to be hospitalized for the 5 days subsequent to evaluation; have telephone access following discharge; and be able to provide consent as measured by the clinical assessment of their healthcare team, the absence of need for a guardian, and a BIMS score of 13 or higher. The four exclusion criteria were being allergic to, or fearful of, dogs; being on contact precautions or being COVID-19-positive; having a BIMS score of 12 or lower; or being the subject of concern by their medical team regarding participation in the study.

Informed Consent: Participants experienced two tiers of written or digital informed consent prior to study participation: (1) screening consent for the inclusion/exclusion criteria assessment, including the BIMS assessment, and, for those meeting study eligibility criteria, (2) providing informed consent for the study.

Participant Incentives: Participants who completed the day 1 measures received a USD 20.00 gift card with additional USD 20 gift cards for those completing each of the 2 follow-up measures. Thus, the total compensation amount for fully participating individuals was USD 60.00 in gift cards.

Phases of the Study: The study was broken into four phases: day 1 (pre-intervention/screening), days 2 to 4 (intervention delivery), day 5 (post-intervention), and longitudinal follow-ups 1 and 6 months later.

Day 1—Consent and Pre-Intervention: At admission, all patients of VCUH are provided the option to indicate whether they are willing to participate in research during their stay. Recruitment personnel for this study only approached patients who responded positively to this question. The informed consent process did include information about the possibility (1 in 3) of interacting with a dog during the study. Further pre-screening was performed by the unit manager, who reviewed the inclusion/exclusion criteria and shared it with the health care staff. These individuals directed the recruitment research assistant to patients who were felt to be good candidates for the BIMS screening. Those patients who cleared that screening reviewed the study procedures with the research assistant, who obtained digital or written informed consent to participate in the study. Day 1 pre-intervention measures were collected (see Figure 1).

Days 2–4—Intervention: Participating patients were randomized into one of the three study conditions, each involving a 20 min intervention repeated on three consecutive days. In all cases, the experimenter stepped outside the room during this 20 min period.

(1) Animal-Assisted Interaction (AAI; n = 15): A Dogs on Call dog and handler team participated in a non-scripted interaction with these participants, a procedure repeated on three consecutive days. To facilitate the fidelity and consistency of treatment delivery, the handler was provided in advance with specific discussion topics, such as sports, the weather, or animals. The same dog/handler team may or may not participate in multiple visits to the same patient. The assignment of dog/handler teams was based purely on their availability.
(2) Conversational Control (CC; n = 15): The handler visited the participant alone, without their dog, for a non-scripted interaction as instructed for the AAI condition. The sessions were also repeated on three consecutive days.
(3) Usual Care (UC; n = 14): No additional interventions were provided to participants in the UC condition beyond their usual hospital care. Participants rested or engaged in a quiet activity during the 20 min intervention period.

Days 2–4—Data Collection: On each of days 2 through 4, the research assistant administered the same pre-session measures at the start of the one-hour session (for the UC group, at a time when no other treatments were being delivered) (see Figure 1). Following the 20 min assigned to each condition (AAI, CC, or UC), the research assistant administered the post-test measures.

Day 5—Post-Intervention: Post-intervention measures (see Figure 1) were administered by the research assistant, who also collected contact information for longitudinal follow-up.

Longitudinal Follow-Up: At 1-month and 6-month intervals, participants were mailed follow-up measures, including a self-addressed, stamped envelope to use in returning their completed forms. Patients were re-contacted by phone once if the measures were not returned. They were asked whether they had received the measures and whether they would be willing to complete them. If so, a new set of measures and return envelope were mailed to them.

***Statistical Analysis:***

Study Satisfaction: A three-group between-subjects ANOVA was used to examine differences in study satisfaction among the groups.

Intervention Period Changes: After checking assumptions, linear mixed models or generalized linear mixed models with random intercepts based on participant identification numbers (IDs) were applied to examine differences in the changes in each outcome from before to after the intervention. Mood was examined with linear mixed models, and anxiety (STAI-AD) and presence of anxiety (dichotomous) were examined with generalized linear mixed models. The analysis employed the generally accepted cut point for the presence of anxiety, a score of 40 or higher [41]. The negative log-log link was used for STAI-AD based on considerable positive skew that could not be corrected by transformation, and the binomial logistic model was used for the presence of anxiety. Predictors in the analysis included the interventions (AAI, CC, and UC), with AAI as the reference group; pre-post, representing the outcome scores before and after the intervention; day, representing the day (1 to 3) of the intervention; and the interactions of the interventions with pre-post and with day. One-tailed tests were used for superiority of AAI to the other interventions based on a priori hypotheses. Sequential Bonferroni corrections were applied.

Study Duration: Baseline to day 5: After checking assumptions, linear mixed models or generalized linear mixed models with random intercepts based on participant IDs were applied to examine differences in the changes in each outcome from baseline to the day after the intervention. Depression and quality of life were examined with linear mixed models, and anxiety was examined with generalized linear mixed models with the negative log-log link. Baseline was compared with post-intervention (Day 5). One-tailed tests were used for superiority of AAI to the other interventions based on a priori hypotheses. Predictors in the analyses included the interventions (AAI, CC, and UC) with AAI as the reference group, the time variable (baseline to day 5), and the interaction of the intervention group with the time variable. All data were included in the analyses.

## 3. Results

### 3.1. Satisfaction

Though not significant, satisfaction scores tended to differ among the groups [*F* (2,21) = 3.197, *p* = 0.061]. Planned comparisons indicated that average ratings differed (*p* = 0.031) between the UC (*M* = 2.80, *SE* = 0.37) and the AAI (*M* = 4.18, *SE* = 0.33) intervention participants but not between the CC (*M* = 3.25, *SE* = 0.45) and AAI groups. The UC and CC conditions were not significantly different from each other. The CONSORT chart illustrates that most participants who withdrew from the study prior to completing the intervention did so due to either worsening illness or early discharge from the hospital. No participants withdrew because they did not want to receive the animal-assisted intervention. Two participants in the control condition withdrew because they were disappointed about not being randomized to the AAI group.

### 3.2. Intervention Period—Mood

**Figure 3:**
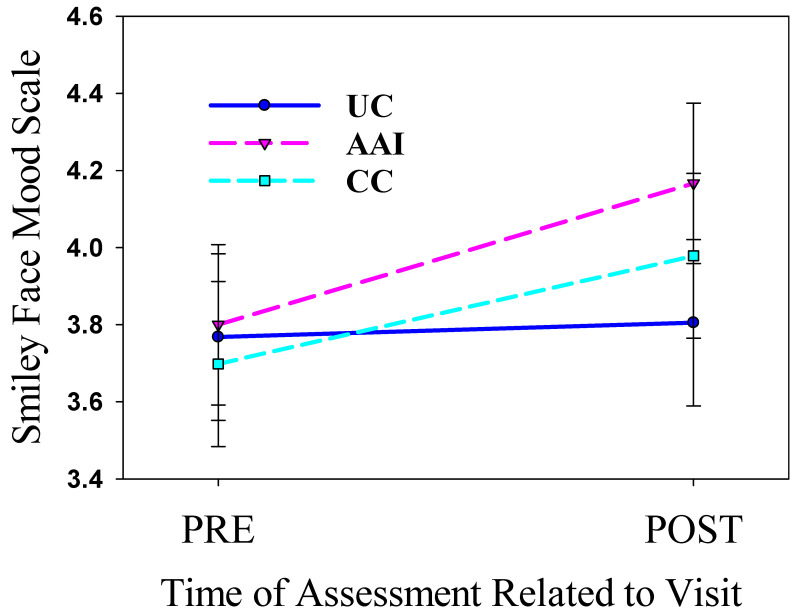
Changes in mood for the animal-assisted intervention (AAI), conversation control (CC) intervention, and usual care (UC) participants from before (Pre) to after (Post) the intervention visits as assessed with the Smiley Face Mood Scale.

Mood improved more for the AAI than for the UC group from before to after the intervention sessions combined [*b* = 0.300, *SE* = 0.164, *p* (one tailed) = 0.034]. Changes in mood from before to after the combined intervention sessions for the CC group were not significantly different [*b* = 0.180, *SE* = 0.169, *p* (one-tailed) = 0.144] than those for the AAI group (see Figure 3). There was no evidence that mood changed or that treatment altered changes in mood from day 1 to day 3 of the intervention segment of the study.

### 3.3. Intervention Period—Amount of Anxiety

There were no significant differences in changes in anxiety scores between the AAI and other groups either from before to after the sessions or between days. Anxiety scores decreased significantly from before to after the intervention sessions for three intervention groups combined (*b* = 0.147, *SE* = 0.069, *p* = 0.036). Anxiety did not decrease significantly from day 1 to day 3 of the intervention segment of the study.

### 3.4. Intervention Period—Presence of Anxiety

**Figure 4:**
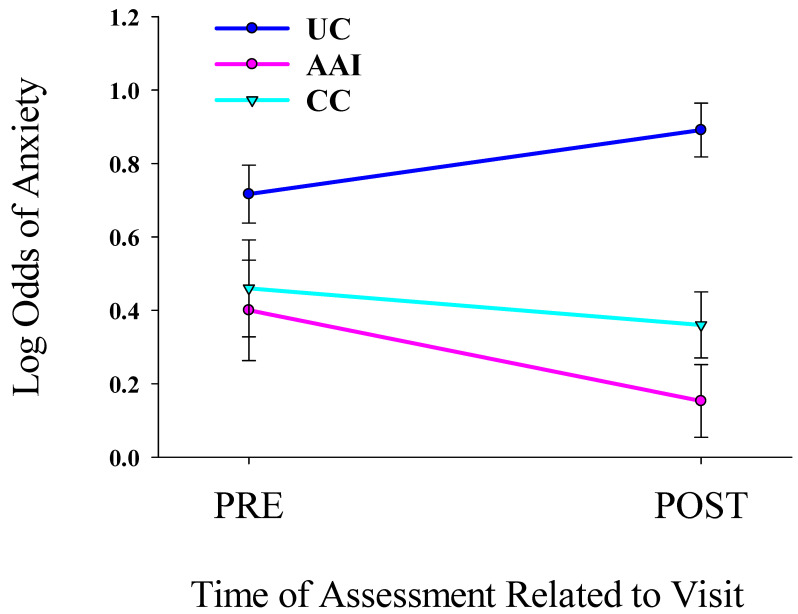
Changes in log odds of the presence of anxiety as assessed with the five item State-Trait Anxiety Inventory for the animal-assisted intervention (AAI), conversation control (CC) intervention, and usual care (UC) participants from before (Pre) to after (Post) the intervention visits.

The odds of anxiety changed differently among the groups from pre- to post- intervention [*F* (2,8) = 13.328, *p* = 0.006]. The presence of anxiety decreased significantly from before to after the 20 min intervention sessions (all three intervention days combined) for the AAI group and not for the CC or UC groups (see Figure 4). There was no evidence that AAI was related to decreases in the odds of anxiety from day 1 to day 3 of the intervention segment (days 2–4 of the study).

### 3.5. Study Duration: Baseline to Day 5 and 1 and 6 Months—Amount of Anxiety

There were no significant differences in changes in anxiety from baseline to the day after the intervention between the AAI and other groups. Anxiety did not differ significantly among the groups or change significantly from baseline to the day after the intervention. There were no significant differences in changes in anxiety from baseline to 1 and 6 months after the intervention between the AAI and other groups. Anxiety did not differ among the groups or change from baseline to 1 and 6 months after the intervention.

### 3.6. Study Duration: Baseline to Day 5 and 1 and 6 Months—Depression

There were no significant differences in changes in depression from baseline to the day after the intervention between the AAI and other groups. Depression was significantly lower overall in the CC group than the AAI group (*b* = 8.353, *SE* = 3.315, *p* = 0.014) and did not change significantly from baseline to the day after the intervention. There were no significant differences in changes in depression from baseline to 1 and 6 months after the intervention between the AAI and other groups. Depression tended to be higher overall (*b* = 4.539, *SE* = 2.512, *p* = 0.077) in the UC group than in the AAI group and did not change significantly from baseline to 1 and 6 months after the intervention.

### 3.7. Study Duration: Baseline to Day 5 and 1 and 6 Months—Quality of Life

There were no significant differences in changes in quality of life from baseline to the day after the intervention between the AAI and other groups. Quality of life did not differ significantly among the intervention groups or change according to time of assessment. There were no significant differences in changes in quality of life from baseline to 1 and 6 months after the intervention between the AAI and other groups. Quality of life did not differ significantly overall among the intervention groups and tended to improve from baseline to 6 months later in the participants (*b* = 0.976, *SE* = 0.539, *p* = 0.077).

## 4. Discussion

This pilot study utilized a randomized controlled design to investigate the effectiveness of a 20 min animal-assisted intervention on depression, mood, and anxiety in hospitalized older adults over a 3-day period with 1- and 6-month follow-ups. Incorporating therapy dog teams with previous experience in an evidence-based animal-assisted intervention program, one with high ethical standards for both humans and canines, enhanced the rigor of the study design. The use of a handler-only control allowed for the segregation of the effects of the dog component of the team, and the inclusion of multiple follow-up assessments introduced the opportunity for the detection of any longitudinal effects. Presence of anxiety and mood showed significant trajectories of change in a positive direction from before the intervention to immediately after the intervention in the AAI condition relative to the UC condition. The trajectories of change in both measures were not significantly different in the CC condition compared to the UC condition. These results indicate that short term interactions with a dog (and their human handler) are effective at reducing anxiety and improving mood for hospitalized older adults. The absence of this finding in the CC condition indicates that the presence of the dog adds value above that of the human handler. These effects, however, were fleeting in this study, because they were not present when comparisons were made from baseline (day 1) to post-intervention (day 5), nor were they present at 1- and 6-month follow-up. This indicates that interactions with a dog do tend to elevate mood and reduce anxiety immediately but may not represent durable effects. Exploring potential physiological and psychological mechanisms underlying older adults’ responses to dogs may shed light on variation in the effects of animal-assisted interventions [46]. In addition, our finding of significant improvements in the binary measure of anxiety (above vs. below clinical cutoff) but not in continuous anxiety measures may reflect specific diagnostic characteristics of our population (having serious, life-threatening health problems) or the clinical context in which the study was conducted (i.e., facing ongoing pain or invasive medical procedures.) However, it is also possible that we did not have the power to detect these effects over time because the size of the effect may be smaller over time than when it is measured immediately. We were unable to meet our recruitment goals in this study because our recruitment rate was extremely low (4.8%) [29]. Thus, the possibility exists that our study was underpowered to detect potentially smaller but durable effects. For example, our sample size was smaller at 1 month (n = 23) and even smaller at 6 months (n = 19) because not all the participants returned their 1- and 6-month measures. In this sample of older adults, it is possible that some surveys were not returned because the participants may have experienced a worsening of their condition, entered residential care, or passed away during the follow-up period.

Depression was not significantly changed by the presence of the dog (AAI) or the presence of the human handler alone (CC) in any of the statistical comparisons. This may indicate, as was discussed earlier, that a longer interaction with the animal is required to move depression scores. This study used a 20 min interaction over the course of three consecutive days, whereas previous research reporting reductions in depression scores due to AAI used longer term interactions, such as 8 weeks [13], 12 weeks [14], and 11 weeks [15]. Depression as a construct may be more intransigent than either anxiety or mood, and thus a longer intervention may be required to make a measurable difference. This possibility remains conjecture at this point, because a non-significant finding can result from many factors including measurement error or not enough power to detect a significant change (e.g., sample size is too small), among others. Nevertheless, it is a point worth investigating in future AAI studies. Likewise, quality of life was not significantly altered by the presence of the dog (AAI) or the human handler (CC), suggesting a similar possibility that it may be a more durable trait, or potentially that a lengthy hospital stay, and the health-related reasons requiring that stay, may be the single largest factor impacting both measures.

Finally, it is interesting to note that study satisfaction scores were significantly higher in the AAI condition than the UC condition. This indicates that hospitalized older adults were more satisfied with the study when they had the opportunity to interact with the dog. It is likely that the participants in this study were individuals who like, appreciate, and value dogs. Therefore, when they were randomized into the dog condition, they were more satisfied about participating in the study. Others who were not randomized into the dog condition may have been disappointed about not having the opportunity to interact with a dog and that disappointment may have presented itself in lower study satisfaction scores. We did not directly assess this, but anecdotally we had participants voluntarily comment about being disappointed that they were not assigned to the dog condition. Given the study description provided in the informed consent process, all participants were aware that the study included the potential to be randomized into a condition that would allow them to interact with a dog. It is possible that people who do not like, appreciate, or value dogs would opt out of participating in the study.

### Limitations

Most limitations to this pilot study result from the recruitment challenges described elsewhere [29]. These challenges resulted in a small sample size that may have underpowered the study and may have produced a convenience sample that is not representative of older adults residing in acute care and rehabilitation facilities generally. Additionally, the opportunity for additional statistical analyses based on demographic subgroups was restricted by the small sample size, which left the subgroups too small for meaningful study.

Some elements of the rigorous study design, such as the experience of the dog-and-handler teams and the standardized protocol time length, may have produced results that are not consistent with what might be expected under different animal assisted intervention circumstances.

As is the case with all research of this nature, people who do not like dogs or are allergic to them would probably not have agreed to participate in the study; therefore, we cannot make any claims about that segment of the population.

## 5. Conclusions

The results of this study indicate that both anxiety and mood are improved, in the short term, in hospitalized older adults who are given the opportunity to interact with a dog and their handler. These effects were present in the AAI compared to UC conditions, but not in the CC compared to UC conditions, indicating that there is something special and unique added to the intervention by the presence of the dog, above and beyond the presence of the human handler. The anxiety- and mood-improving effects reported in this study, however, were not durable, in that they were not apparent in any of the longer-term comparisons. Depression scores were also not significantly changed in this study, suggesting, among other things, the possibility that depression is a more intransigent construct requiring a longer intervention in order to effect change in those scores. Findings of even temporary improvements in mood and anxiety may offer valuable means for improving the daily experience for older adult inpatients, which in turn could improve compliance with treatment or potentially reduce pain. These possible impacts are worth exploring in future research. From a scientific perspective, these findings offer useful information about the types and durability of outcomes that can be impacted by AAI. We suggest that future research consider that AAI may play a mitigating role in the way length of stay and severity of illness/trauma impact quality of life and/or depression in hospitalized patients.

## Data Availability

The data are publicly available at Open Science Framework and can be accessed via this link: https://osf.io/4ca9b/files/osfstorage/684ed53595b5363cc2cc2b3a.

## Abbreviations

The following abbreviations are used in this manuscript:

AAI	Animal-Assisted Intervention
CC	Conversational Control condition—Handler Only
UC	Usual Care Condition
DoC	Dogs on Call
VCU	Virginia Commonwealth University
VCUH	Virginia Commonwealth University Health System
BIMS	Brief Interview for Mental Status
CESD	Center for Epidemiological Studies, Depression
STAI	State Trait Anxiety Inventory
HRQOL	Health Related Quality of Life
